# A Trans-pedicle Approach to Avoid Exiting Nerve Root Injury in Full-Endoscopic Transforaminal Decompression: A Case Report

**DOI:** 10.7759/cureus.43573

**Published:** 2023-08-16

**Authors:** Dawei Guan, Caitlin M Gray, Shreya Aggarwal, Sanjeev Kumar

**Affiliations:** 1 Department of Pharmaceutical Outcomes & Policy, University of Florida College of Pharmacy, Gainesville, USA; 2 Division of Pain Medicine, Department of Anesthesiology, University of Florida College of Medicine, Gainesville, USA; 3 Department of Anesthesiology, North Florida/South Georgia Veterans Health System, Gainesville, USA

**Keywords:** trans-pedicle approach, foraminotomy, transforaminal stenosis, surgical technique, endoscopic spine decompression

## Abstract

This report aims to describe a case with an extremely low-located exiting nerve root and introduce the trans-pedicle approach for endoscopic transforaminal decompression, which can enable the safe visualization of the exiting nerve root. We present the medical history, imaging findings, and surgical procedures of a 62-year-old male who underwent left L3/4 and L4/5 endoscopic transforaminal decompression for foraminal stenosis.

The patient presented with pain and numbness in the anterior and lateral aspects of the left thigh. MRI revealed left L3/4 and L4/5 foramen stenosis and endoscopic transforaminal decompression was performed. The working channel was guided and positioned at the upper lateral part of the left L4 pedicle. We observed the L3 root being compressed by scar tissues against the upper edge of the L4 pedicle. Then we used a protective diamond burr to drill the upper part of the L4 pedicle, enlarging the L3/4 foramen. Subsequently, pituitary Rongeur, Kerrison, and punches were employed to meticulously remove scar tissues around the exiting root. The same procedure was performed for left L4-5 foramen decompression. The radicular symptoms were relieved immediately after the surgery.

Our proposed trans-pedicle endoscopic transforaminal approach can reduce the risk of injuring a low-located exiting nerve root. It can also help standardize the procedure, improves working channel stability, and facilitates the learning process, making it a valuable technique for full-endoscopic transforaminal decompression.

## Introduction

Endoscopic transforaminal decompression is a promising technique because of its minimal invasion, better visualization of pathology, and shorter recovery time [1]. However, one notable concern associated with the full-endoscopic approach is the potential risk of nerve root injury [2]. In a systematic review conducted by Yin et al., it was reported that the risk of nerve root injury in the percutaneous full-endoscopic transforaminal approach was approximately 2% [3].

In a typical endoscopic transforaminal approach, the guide wire is directed to land at the Kambin’s triangle, followed by the placement of working channels [4]. Kambin’s triangle, positioned below the exiting nerve and over the dorsolateral disc, is widely recognized as a safe corridor for the endoscopic transforaminal approach [5,6]. Chen et al. employed MRI to measure the distance between the exiting roots and the Kambin’s triangle in patients without previous lumbar surgeries, concluding that it provides a safe route for the endoscopic transforaminal approach [7].

However, when the exiting nerve root is positioned low in the foramen, there is a significant risk of nerve injury due to the non-visualized approach. In this regard, we present a case with an extremely low-located exiting nerve root and introduce a technique that enables safe visualization of the nerve root: the trans-pedicle approach for full-endoscopic transforaminal decompression. By docking on the lateral part of the lower pedicle, this approach provides the advantage of visualizing the exiting nerve root before entering the foramen. As a result, the risk of exiting nerve root injury is significantly reduced.

## Case presentation

**Figure 1:**
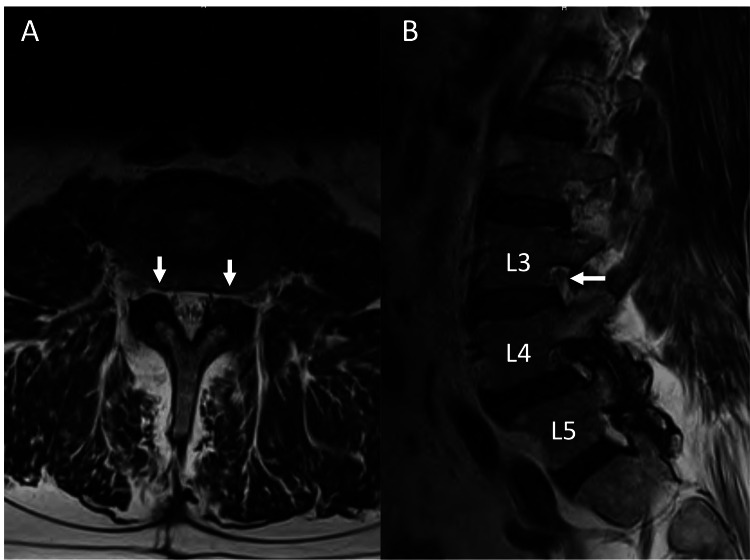
Pre-operation MRI. A: Axial view demonstrated bilateral neuroforaminal stenosis at the L3/4 level (arrows). The radicular symptom was more severe on the left side and we performed left full-endoscopic transforminal decompression. B: Sagittal view revealed that the left L3 exiting nerve root appears to be high in the L3/4 foramen (arrow). During the surgery, the L3 exiting root turned out to be low. This emphasizes that we could not purely rely on MRI to determine the position of the nerve root.

A 62-year-old male patient received L4/5 laminectomy with partial facetectomy six years ago for radiculopathy and neurogenic claudication. This year, the patient presented to the clinic with pain and numbness in the anterior and lateral aspects of the left thigh. MRI indicated bilateral L3/4 and L4/5 foramen stenosis (Figure 1).

On the MRI, the left L3 root appeared to be high in the foramen. As the radicular symptom was more severe on the left side, transforaminal endoscopic decompression of left L3/4 and L4/5 foramina was performed. The following narrative provides a summary of the decompression process.

**Figure 2:**
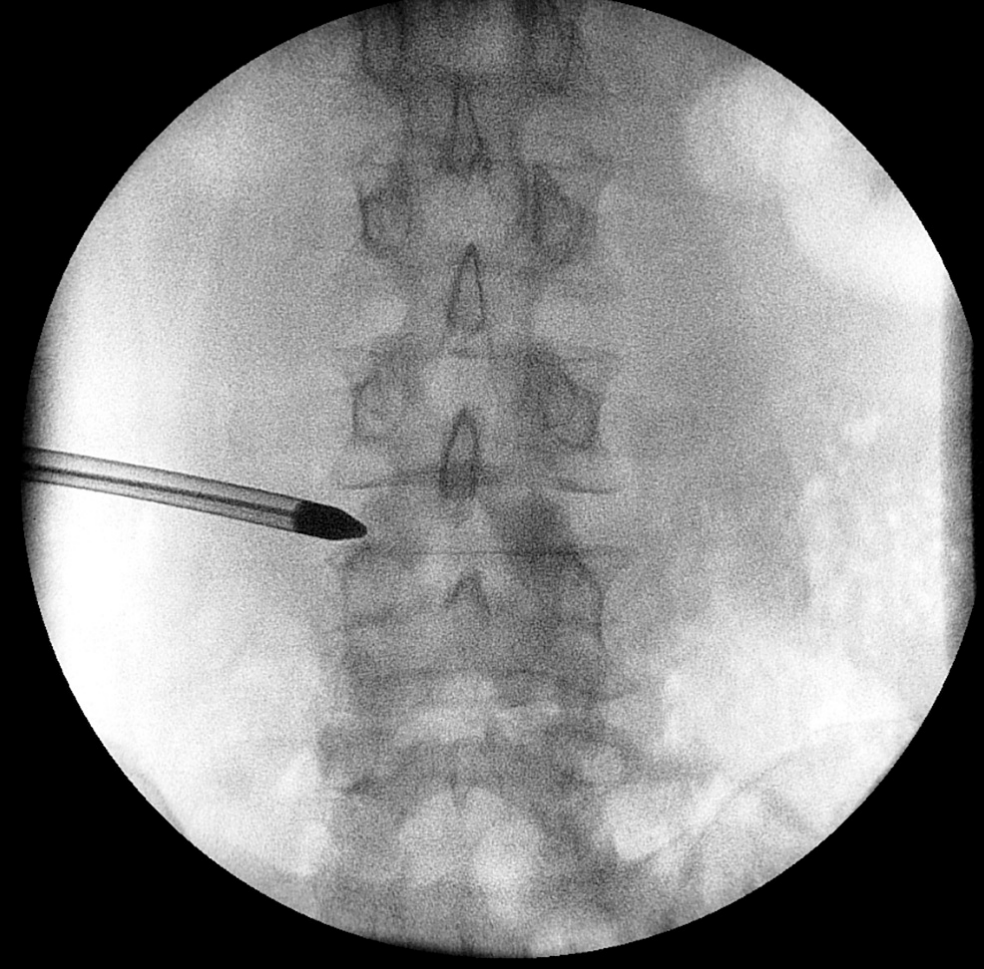
Fluoroscopy. Fluoroscopy demonstrates the initial approach positioned at the lateral upper part of the L4 pedicle.

The patient was under general anesthesia and in a prone position on a Wilson frame. A 16-gauge needle was introduced to the lateral upper part of the left L4 pedicle, guided by fluoroscopy. Following dilation, the working channel and endoscopy were carefully positioned at the lateral part of the L4 pedicle (Figure 2).

**Figure 3:**
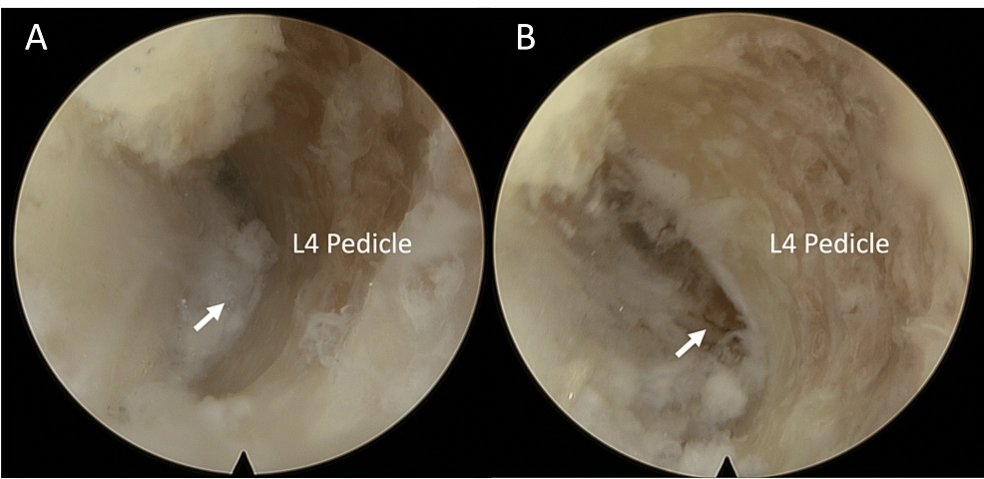
Endoscopic view. A: Endoscopic view of left L3/4 foramen before visualizing the exiting root. Arrow shows a narrow space in the left L3/4 foramen. B: Exposing the left L3 exiting root after drilling the upper edge of the L4 pedicle. Arrow shows the left L3 root and its accompanying blood vessels. Note the small space between the exiting root and pedicle.

We used bipolar radiofrequency cautery and pituitary Rongeur to remove the soft tissue and visualize the lateral upper part of the left L4 pedicle. During this process, the neurological monitor sounded an alarm and we observed the L3 root being compressed by scar tissues against the upper edge of the L4 pedicle. Then we used a protective diamond burr to drill the upper part of the L4 pedicle to enlarge the L3/4 foramen (Figure 3).

Following access to the L3/4 foramen, we proceeded to employ pituitary Rongeur, Kerrison, and punches to meticulously remove scar tissues around the exiting root. The same procedure was employed for the left L4/5 foramen decompression.

## Discussion

During the surgery, the left L3 exiting nerve root was found to be significantly lower than its apparent position on the pre-operation MRI scan, highlighting the limitation of relying solely on MRI for accurately assessing the location of the exiting nerve root within the foramen. Several factors might contribute to the low position of the left L3 exiting nerve root. First, the patient received an L4/5 laminectomy six years ago, resulting in the presence of scar tissue and adhesions, which limited the mobility of nerve roots in adjacent segments. Second, the patient was positioned on a Wilson frame during the surgery, with the lumbar segment in a flexed position. In contrast, the MRI was performed in a supine position with an extended lumbar spine. The combination of (1) the elimination of lower lumbar lordosis during the surgery and (2) a relatively fixed nerve root secondary to epidural scarring from the prior surgery brought the exiting L3 nerve to a lower position in the left L3-4 foramen.

When performing the full-endoscopic transforaminal approach, special caution should be exercised in cases with a history of spine surgery. In our proposed approach, we use the upper lateral pedicle as a reliable landmark for guiding the working tube, ensuring direct visualization of Kambin’s triangle before entering the foramen. If a low-located exiting nerve root is encountered, a protective burr will be used to drill the upper edge of the pedicle to access the foramen. By docking on the bone, this approach can significantly reduce the risk of exiting nerve root injury. A similar strategy was used by Greil et al., who utilized the pars interarticularis as a docking point to address the far-out syndrome [2].

This case further emphasizes the value of neurological monitoring in preventing nerve injury during endoscopic decompression under general anesthesia. In situations where neurological monitoring is unavailable, it is advisable to perform the surgery under local anesthesia with the patient awake, which allows the patient to provide feedback if the nerve is irritated.

Besides reducing the risk of nerve root injury, our approach offers additional benefits. First, by using the bony landmark at the lower pedicle, the process can be easily standardized, particularly for revision surgeries. Yagi et al. reported variations in the accessing process depending on the severity of foraminal stenosis in revision surgeries [8]. In contrast, our proposed trans-pedicle approach demonstrates less variation and is easily applicable across cases. Second, landing on the pedicle aids in stabilizing the working channel, thereby further reducing the risk of inadvertent nerve injury. Third, our proposed approach is easy to apply, which can help flatten the learning curve. One of the main challenges when learning full-endoscopic transforaminal decompression is understanding the anatomy around the approaching path and under the scope [9]. Our method provides surgeons with a sense of safety when accessing the foramen, making the transforaminal approach more manageable. However, caution should be taken when performing this approach. Drilling of the upper edge of the pedicle should be minimized to a necessary extent. Excessive drilling may pose a risk of spine instability in the future.

## Conclusions

Our proposed trans-pedicle approach offers the advantage of visualizing the exiting root before entering the foramen, effectively minimizing the risk of an inadvertent nerve injury. Additionally, this technique uses the lower-level pedicle as a consistent landing mark, making it easily standardized during training and facilitating the learning process. Therefore, we highly recommend adopting this approach as the standard practice for endoscopic transforaminal decompression procedures.
